# A Litopenaeus vannamei shrimp dataset for artificial intelligence-based biomass estimation and organism detection algorithms

**DOI:** 10.1016/j.dib.2024.110964

**Published:** 2024-09-26

**Authors:** Fernando J. Ramírez-Coronel, Edgard Esquer-Miranda, Oscar M. Rodríguez-Elias, Pedro García-Hinostro, Guadalupe César Parra-Salazar

**Affiliations:** a Tecnológico Nacional de México / I.T. de Hermosillo, Division of Graduate Studies and Research, Av. Tecnológico 115, Hermosillo, Sonora C.P. 83170, México; b Universidad Estatal de Sonora, Av. Ley Federal del Trabajo S/N, Hermosillo, Sonora C.P. 83100, México; c Tecnológico Nacional de México / I. T. de Guaymas, Avenida Tecnológico Km.4.0 sector playitas. Guaymas, Sonora C.P 85480, México

**Keywords:** Computer vision, Machine learning, Aquaculture, Allometry, Shrimp

## Abstract

Pond biomass estimation and non-invasive biometrics are necessary problems to solve in shrimp farming to achieve the optimization of currently outdated manual processes which are slow, inaccurate, imprecise, and prone to errors. This dataset was collected to develop and test computer vision and artificial intelligence models to accurately detect shrimps and estimate their biomass. The dataset was collected in three ponds, two in an industrial farm and the other in a university pond cultivated for academic purposes. 170 shrimps were sampled by taking pictures and manual measurements of their total length, cephalothorax length, and weight. A total of 5507 shrimp’ images were taken which were put in containers equipped with cameras and with a water level of 10 cm. The dataset is organized into five sub-datasets folders and excel files containing the manual measurements taken with a scale and vernier from each sample. This dataset could be used to compare and develop different detection and biomass estimation computer vision models since it presents a good amount of images and samples of shrimps cultivated in different conditions which can allow models to relate image features of shrimp samples with their corresponding weight and also compare these models against the machine learning models that can be applied solely to the manually extracted features stored in the excel files for biomass estimation.

Specifications Table

**Table:** 

Subject	Computer Vision and Pattern Recognition
Specific subject area	Computer vision, machine learning, biomass estimation in aquaculture farms, and morphology and allometry computer vision aided studies.
Type of data	Table (excel files), Image (png images).
Data collection	Photos were taken from shrimps collocated on containers with 10 cm of water. Two different cameras were located at two ice containers, a Logitech c920 connected to a laptop and raspberry pi HD camera of 12 Mp connected to a raspberry pi b3 model. For each shrimp more than 20 images were taken with a time interval per image of 1 to 2 s (the time parameter and number of pictures per shrimp was varied in the practice to look for better results), the software was programmed using Python, OpenCV, Pillow and Glob library. The conditions of data collection varied in source and conditions for different groups.
Data source location	First part of dataset • Industrial Shrimp farm located in Cardenal Hermosillo • Hermosillo, Sonora - 83317 • México • 28.522099, -111.627426 Second part of dataset: • Academic pond located in Instituto Tecnológico de Guaymas • Guaymas, Sonora - 85480 • México • 27.894884, -110.893038
Data accessibility	Repository name: Mendeley Data Data identification number: 10.17632/h8tcn6ykky.1 Direct URL to data: https://data.mendeley.com/datasets/h8tcn6ykky/1 [1] Instructions for accessing these data: Click on the provided link, enter it into your browser, or search using the DOI identifier

## 1 Value of the Data

• The dataset is the only one known open access dataset of shrimp (*Litopenaeus vannamei*) having a considerable number of images and shrimps, 5507 images, and 170 shrimp samples, taken in industrial and academic ponds with their related manually measured data (total length, cephalothorax length, and weight) in an Excel file which could allow aquaculturists, biologists, machine learning engineers, and researchers in general, to develop and test new methods for biomass estimation enhancing precision and understanding in aquaculture and biology.
• Researchers can use this data to develop and test computer vision and machine learning methodologies for shrimp biomass estimation. It could also be used to investigate by using statistical analysis, the allometric relationships of extracted shrimp features with their biomass and morphology in general.
• The images could also be specifically used to train object detection models for shrimp in real time.
• The research gap addressed by this dataset is the development and validation of methods for estimating shrimp biomass by relating specific image features of shrimp to their biomass. One example is the work done by [2], where regression functions were developed to relate the maximum eye length of shrimp to their total biomass. Another example is the work done by [3], which used eight selected morphometric features to estimate biomass. Therefore, this dataset, being unique in size and data—at least to the author's knowledge—can help establish a common ground for developing, testing and comparing various methodologies.

## 2 Background

Shrimp biomass precise estimation is essential for aquaculture industries [4]. First, it helps to know how much food to be given to the shrimps, which is the principal operational cost in aquaculture farms [5]. Feed management is important not only for economic purposes but also for environmental care [6]. It is also important for stakeholders to know the number of shrimps and total pond biomass to make business. The methods currently used for getting this information are invasive, imprecise, and slow which can stress and cause animal deaths [7]. Better methods for biomass estimation and non-invasive biometrics are necessary and in line with the advancing field of precision aquaculture, which applies control concepts to aquaculture for optimizing production [8]. With this precision farming philosophy in mind, this dataset was collected to include multiple photos of shrimps, their weight, and length measurements with the end goal of developing better non-invasive biomass estimation methods based on computer vision and artificial intelligence algorithms for the aquaculture industry sector, as well as for academic studies of shrimp development and allometry.

## 3 Data Description

**Fig. 1:**
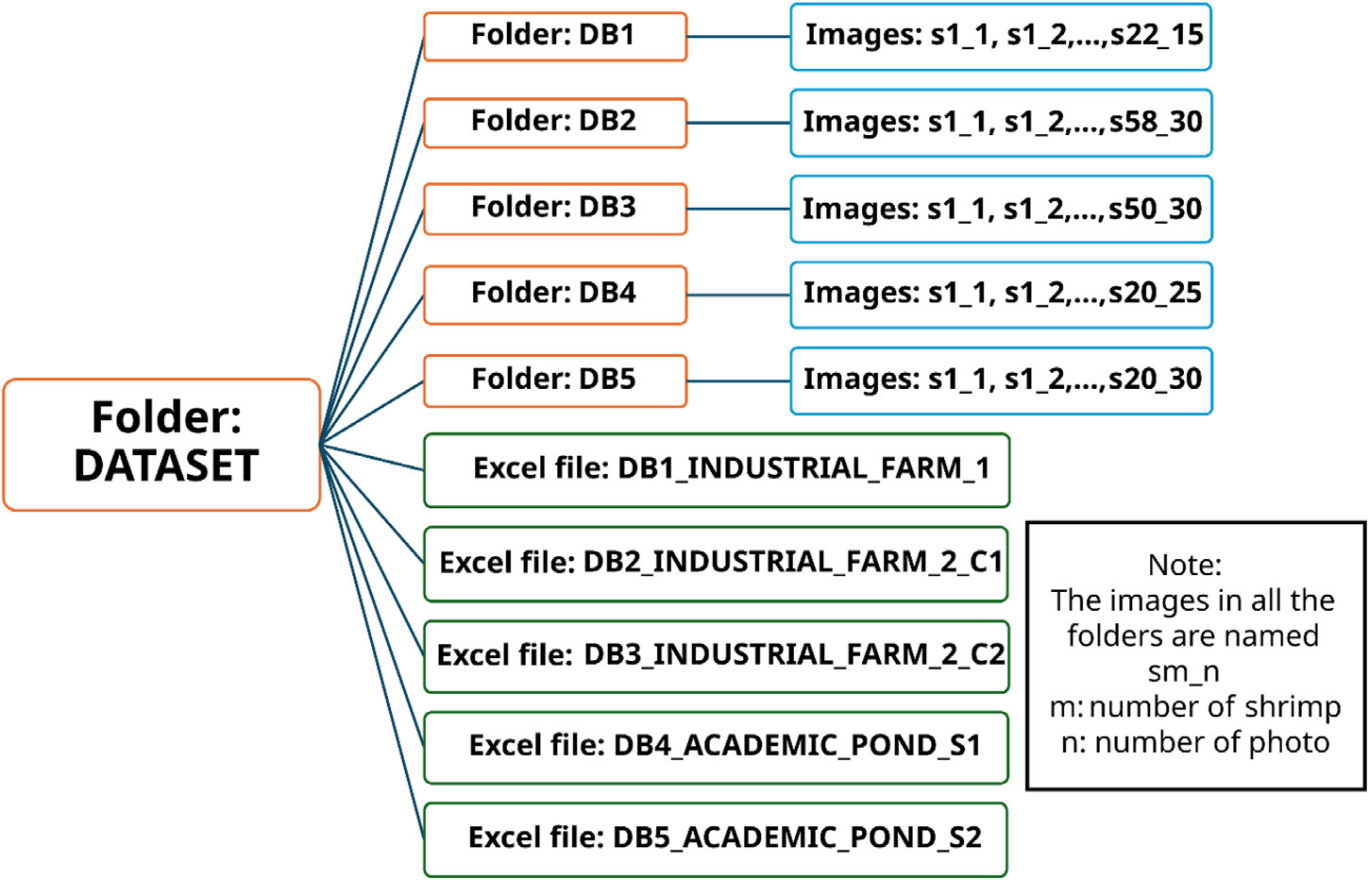
Dataset's folders and files structure.

sThe dataset in [1] is divided into 5 folders containing a total of 5507 images from 170 sampled shrimps and corresponding excel files containing the manual measurements taken from each of the shrimps. The structure of the dataset files is in Fig. 1, which shows the organization of the folders, excel files, and images. The images have a resolution of 640×480 pixels and are in PNG format.

The name of the shrimp follows the same logic for all the folders as it is explained in the next paragraph and mentioned as a note in Fig. 1. The sub datasets were created from shrimps of three ponds, two industrial shrimp farm ponds and one university pond for academic purposes.

**Fig. 2:**
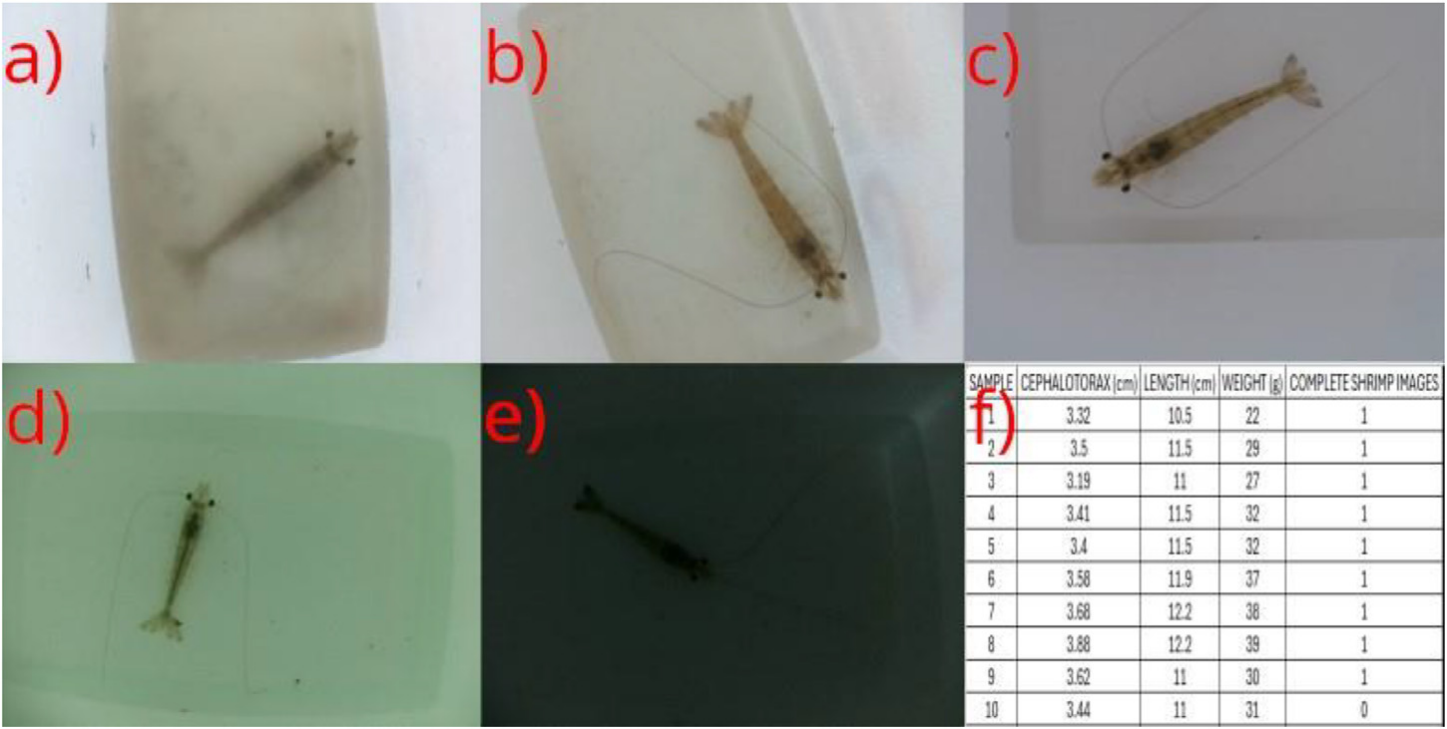
Example images of the different sub-datasets. Images of shrimps (a) to (e) and images of the manually measured data (f).

The first folder, “DB1”, contains the images taken from the first capturing process at the industrial farms. The images are referred to as “**sm_n”** where s refers to shrimp, m refers to the shrimp number, and n to the image number of that shrimp. This folder encompasses 490 images of 22 shrimps. Its corresponding excel file “DB1_INDUSTRIAL_FARM_1” contains the following columns for the 22 shrimps: “SAMPLE”, “LENGTH (cm)”, “WEIGHT (g)” and “COMPLETE SHRIMP IMAGES” (if 1 there is at least one image with the full body of the shrimp). An example of an image of this sub-dataset is shown in Fig. 2a.

The second folder, “DB2”, contains the images taken from the second capturing process in the second pond of the industrial farm using the container. This folder encompasses 2002 images of 58 shrimps. Its corresponding excel file “DB2_INDUSTRIAL_FARM_2_C1” contains the following columns for the 58 shrimps: “SAMPLE”, “CEPHALOTHORAX (cm)”, “LENGTH (cm)”, “WEIGHT (g)” and “COMPLETE SHRIMP IMAGES” (1 if there is at least one image with the full body of the shrimp). An example of an image of this sub-dataset is shown in Fig. 2b.

The third folder, “DB3”, contains the images taken from the second capturing process using the second container in the second pond of the industrial farm. This folder encompasses 1719 images of 50 shrimps. Its corresponding excel file “DB3_INDUSTRIAL_FARM_2_C2” contains the following columns for the 50 shrimps: “SAMPLE”, “CEPHALOTHORAX (cm)”, “LENGTH (cm)”, “WEIGHT (g)” and “COMPLETE SHRIMP IMAGES” (1 if there is at least one image with the full body of the shrimp). An example of an image of this sub-dataset is shown in Fig. 2c.

The fourth folder, “DB4”, contains the images taken from the first capturing process in the academic pond. This folder encompasses 635 images of 20 shrimps. Its corresponding excel file DB4_ACADEMIC_POND_S1 contains the following columns for the 20 shrimps: “SAMPLE”, “CEPHALOTHORAX (cm)”, “LENGTH (cm)”, “WEIGHT (g)” and “COMPLETE SHRIMP IMAGES” (1 if there is at least one image with the full body of the shrimp). An example of an image of this sub-dataset is shown in Fig. 2d.

The fifth folder, “DB5”, contains the images taken from the second capturing process in the academic pond. This folder encompasses 661 images of 20 shrimps. Its corresponding excel file “DB5_ACADEMIC_POND_S2” contains the following columns for the 20 shrimps: “SAMPLE”, “CEPHALOTHORAX (cm)”, “LENGTH (cm)”, “WEIGHT (g)” and “COMPLETE SHRIMP IMAGES” (1 if there is at least one image with the full body of the shrimp). An example of an image of this sub-dataset is shown in Fig. 2e.

Fig. 2f shows a capture of one of the excel files, which encompasses columns representing the measured characteristics and a final column indicating whether there is at least one image of the sample where the shrimp is shown in full body. This is because some of the shrimps remained in a corner during the entire image capturing process.

In Table 1, an explanation of excel file columns is provided and in Table 2, the first three samples of “DB1” are presented showing 4 columns, and three samples of “DB3” are presented showing 5 columns (the same as “DB2”, “DB4” and “DB5”). To clarify even more, if a researcher examines sample 1 of “DB1” in Table 1 she will see the first shrimp sampled of the sub-dataset “DB1” which is 12.2 cm in length, weights 33 g, and has images that show its full body. If she examines sample 2 of “DB3”, she will see the second shrimp sampled for sub-dataset “DB3”, which has a corresponding cephalothorax length of 3.69 cm, a total length of 11.5 cm, a weight of 31 g but does not have an image showing its full body.

**Table 1:** Excel files columns description.

Columns	Description
SAMPLE	The sample number in that particular dataset.
CEPHALOTHORAX (cm)	The length measurement of the shrimp at the cephalothorax. The length of the cephalothorax was measured from the base of the rostrum at the eye level to the point where the shrimp's ostium is located, where it connects with the first segment of the abdomen.
LENGTH (cm)	The total length of the shrimp is measured from the rostrum at eye level to the base of the tail.
WEIGHT (g)	The weight measured in grams on a scale.
COMPLETE SHRIMP IMAGES	Indicates if there is at least one image showing the full shrimp for that sample. 1 - Yes, 0 - No.

**Table 2:** Samples of DB1 and DB3 excel files.

Samples of DB1				
SAMPLE	LENGTH (cm)	WEIGHT (g)	COMPLETE SHRIMP IMAGES	
1	12.2	33	1	
2	14.4	33	1	
3	12.24	31	1	

**Samples of DB3**				
SAMPLE	CEPHALOTHORAX (cm)	LENGTH (cm)	WEIGHT (g)	COMPLETE SHRIMP IMAGES

1	3.84	12.5	37	1
2	3.64	11.5	31	0
3	3.59	11.5	36.5	1

**Fig. 3:**
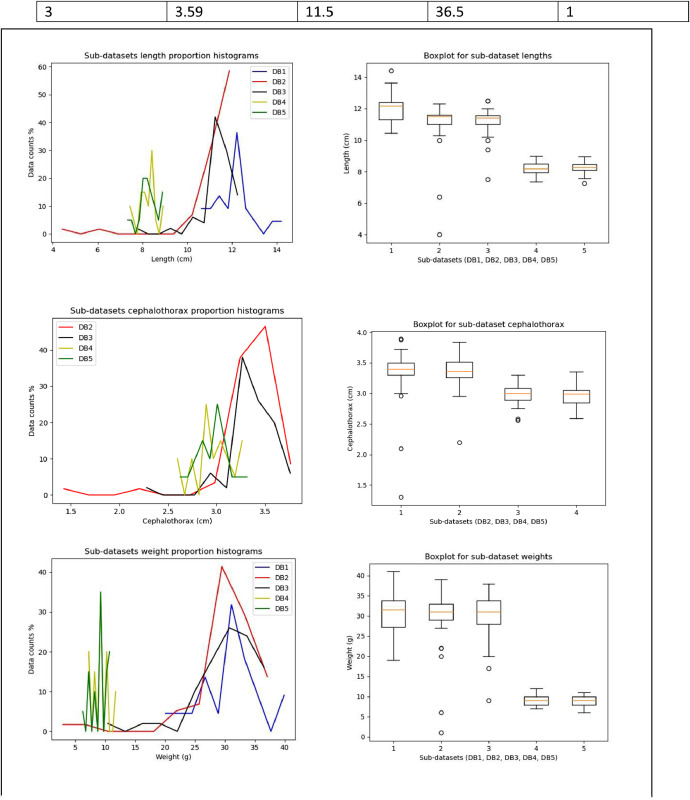
Histograms and boxplots for the different sub-datasets variables (Total length, cephalothorax length, weight).

Fig. 3 shows histograms and boxplot graphs for the sub-datasets for all the variables: total length, cephalothorax length, and weight. It aids to show the difference in ranges of size for the different sub-datasets. It also gives an idea of the form of the data distributions. The cephalothorax graphs show the distribution of measurements for four of the five sub-datasets, as cephalothorax measurements were not taken during the first biometric collection, “DB1”. The boxplot graphs indicate that DB1, DB2 and DB3 have more clearly visible outliers compared to “DB4” and “DB5”. This can be explained by the fact that the industrial farms introduced new baby shrimps into the ponds when the shrimps were about four months old. So, there were shrimps three months younger than the older ones, which was reflected in the size and weight measurements. This did not occur with “DB4” and “DB5” where all the shrimps were of the same age. The graphs clearly show that the median size and range of sizes of the shrimps in “DB1”, “DB2”, and “DB3” are larger than those of the shrimps in “DB4” and “DB5”.

## 4 Experimental Design, Materials and Methods

**Fig. 4:**
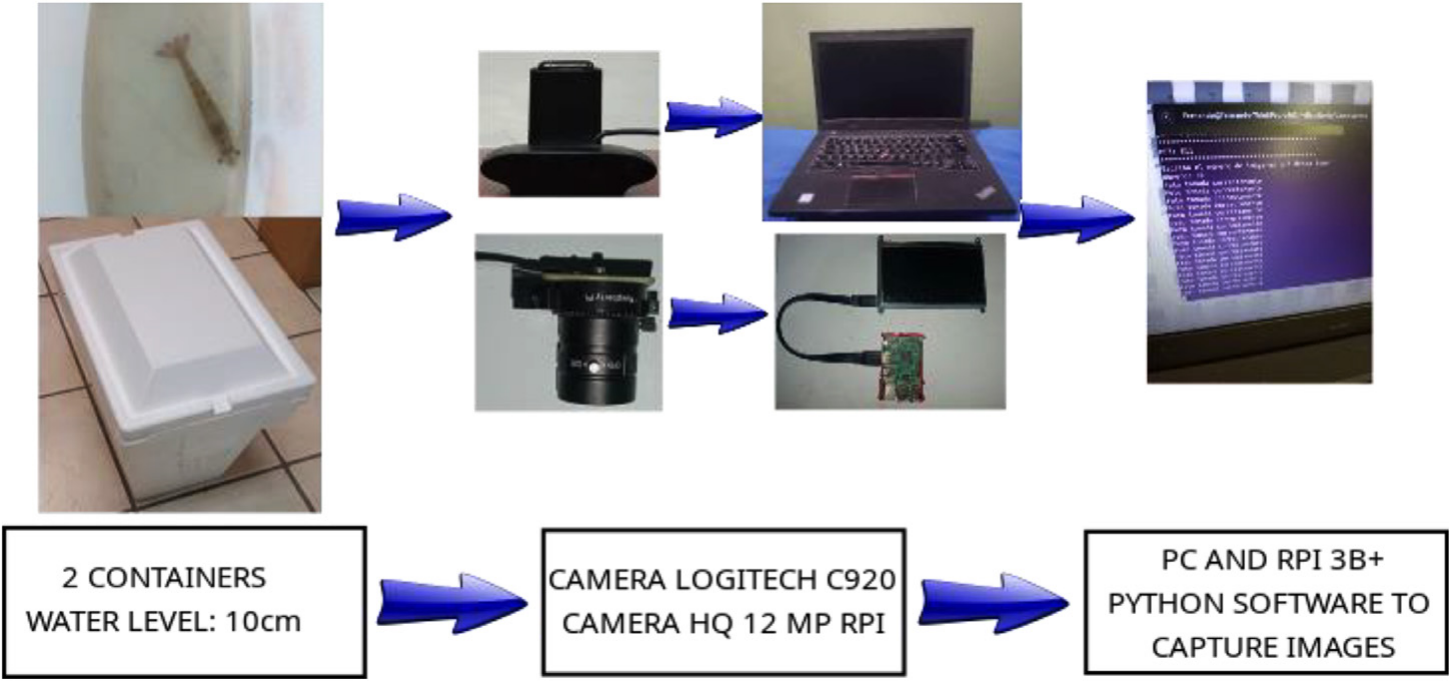
Hardware diagram of the image capturing process.

**Fig. 5:**
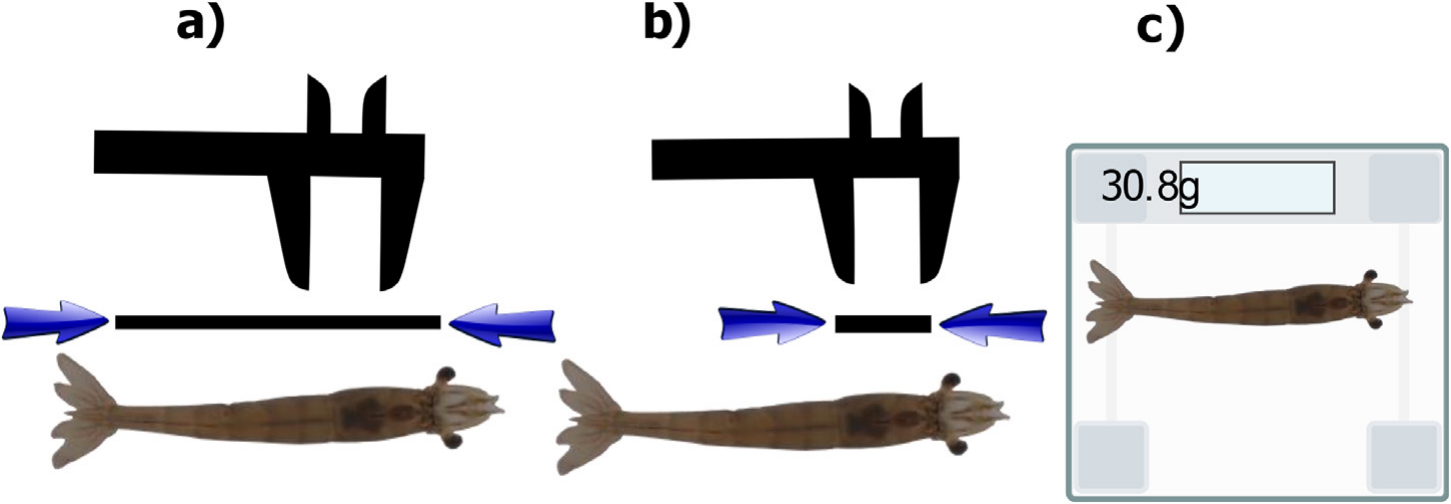
Manual measurement process. (a) Length (cm), (b) Cephalothorax (cm), and (c) weight (g).

**Fig. 6:**
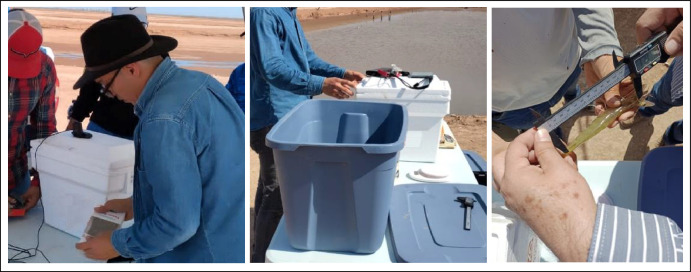
Setups for image capturing processes and shrimp biometrics data extraction.

The dataset was acquired using two cameras installed in containers. The cameras are a high-resolution Raspberry Pi camera of 12MP with panoramic lenses of 3mm and a Logitech c920 webcam. The hardware diagram for both setups is shown in Fig. 4. The ice containers were filled with water until it reached 10 cm. The idea behind this was to capture shrimps in their normal position seen from above and to capture, if possible, photos in different positions and orientations given by their movement in the container. The size and weight of the shrimps were measured using a caliper and a scale. For datasets “DB1”, “DB2”, and “DB3” the shrimps were alive when the images were captured. For “DB4”, some shrimps were alive when the images and measurements were taken. For “DB5”, all the shrimps were already dead when measurements were taken. The aforementioned circumstances of the capturing process were determined by the institutions that provided the shrimps. In other words, the authors adhered to the farming periods and received the shrimps (alive, dead, healthy, etc.) as they were provided during the farming process. The shrimps were being farmed, so they were placed into a container; from where they were extracted one by one and put into another container equipped with the camera, then a user put the number of photos and let the system capture the images. After the photography part was finished, the shrimp was measured using a caliper and weighted using a scale as shown in Fig. 5. Fig. 5a shows how the length was measured (from the rostrum at eye level to the base of the tail), Fig. 5b shows that the cephalothorax length was measured (length of the cephalothorax as stated in Table 1 and Fig. 5c shows how the weight was measured (the shrimp was positioned on the scale). The real setup is shown in Fig. 6 where the images and manual measurements were taken at the industrial farm. The middle image from Fig. 6 shows the gray container with some shrimps, which then were placed one by one into the container equipped with the camera. Then the shrimp after being manually measured with caliper and scale was put into other gray container to avoid confusing with the non-measured shrimps to be farmed afterwards. This process was repeated for each shrimp.

**Fig. 7:**
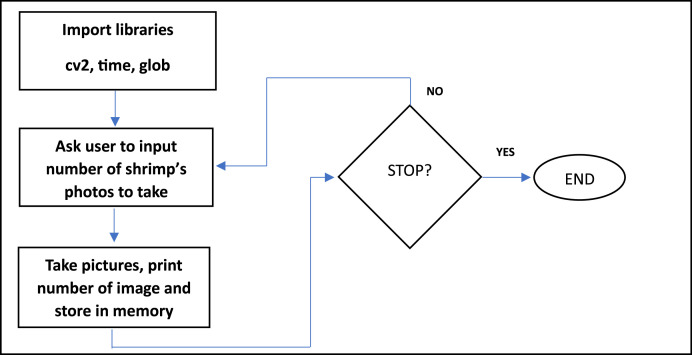
Program for dataset creation.

The program used to capture the shrimp images was made in Python using OpenCV, time and Glob libraries. A simplified version of the program is shown in Fig. 7.

The samples were taken from two different locations and measured in different places and light conditions. The shrimps were randomly extracted from three different ponds (two industrial ponds and one academic pond). Each of the dataset generation processes is described as follows:

The sub-dataset “DB1” and its corresponding excel file “DB1_INDUSTRIAL_FARM_1” were generated at the shrimp farm around noon using the Raspberry Pi 3b and the HQ 12 Mp camera. The shrimps were sampled from one pond while they were being farmed.

The sub-dataset “DB2” and its corresponding excel file “DB2_INDUSTRIAL_FARM_2_C1” were generated at the shrimp farm around one week later at noon using the Raspberry Pi 3b and the HQ 12 MP camera installed in the corresponding container. The shrimps were sampled from a different pond than “DB1” before being farmed.

The sub-dataset “DB3” and its corresponding excel file “DB3_INDUSTRIAL_FARM_2_C2” were generated in the shrimp farm around the same time than the sub dataset “DB2” using a Logitech c920 camera installed in the corresponding container. The shrimps were sampled from the same pond than “DB2” before being farmed.

The sub-dataset “DB4” and its corresponding excel file “DB4_ACADEMIC_POND_S1” were generated at a university's pond in laboratory light conditions using the Raspberry Pi 3b and the HQ 12 MP camera. The shrimps were sampled before being farmed. Most of these shrimps were already dead when capturing the photos.

The sub dataset “DB5” and its corresponding excel file “DB5_ACADEMIC_POND_S2” were generated in a specific location (non-laboratory, with environmental lighting conditions) using the farmed shrimps obtained at the university's pond, with the Raspberry Pi 3b and the HQ 12 MP camera. They were all already dead, so they were carefully positioned to capture their upper body. They were measured and photographed at night under poor lighting conditions due to the environmental setting. They were extracted from the same pond than the shrimps of the “DB4” sub-dataset.

The weather during the sampling processes of “DB1”, “DB2”, “DB3”, and “DB4” was sunny. The images for “DB5” were captured at night. For the sub-datasets “DB2” to “DB5” clear sea water was put in the camera-equipped container. In the case of “DB1” which was the first captured sub-dataset, pond water was used.

In the final stages of the sampling process for DB2 and DB3, which were created simultaneously, due to time restrictions, the authors had to choose randomly extracted shrimps from the ponds placed in the gray container for sampling. To add more variability to the dataset, the authors chose shrimps with smaller size than the average.

## Limitations

This dataset does not encompass shrimps from all the cultivation periods. It includes shrimps from three different ponds (two industrial ponds and one academic pond). Sub-dataset DB1 was captured using pond water, which may introduce turbidity affecting image processing. Sub-dataset DB5 was captured under nighttime lighting conditions. Sub-datasets DB4 and DB5 consist of shrimps that were already dead, so they do not present shrimps with the same variability in position and orientation as DB1, DB2, and DB3. The final selection of specimens for DB2 and DB3 could introduce some skewness or noise to the dataset, as it was influenced by the authors' choices to add variability in the measurements.

## Ethics Statement

The experiments were complied with the ARRIVE guidelines and were carried out in accordance with the U.K. Animals (Scientific Procedures) Act, 1986 and associated guidelines; EU Directive 2010/63/EU for animal experiments; or the National Institutes of Health guide for the care and use of laboratory animals (NIH Publications No. 8023, revised 1978).

The care and use of experimental animals complied with the articles 8, 30 and 35 of “LEY GENERAL DE VIDA SILVESTRE” of “los Estados Unidos Mexicanos” with DOF 20-05-2021 and given what is stablished in article 8 of the aforementioned law, the processes were carried out according to the principles of the “LEY DE PROTECCIÓN A LOS ANIMALES PARA EL ESTADO DE SONORA” (law of protection of animals of the state of Sonora) and meets the requirements of the 17th article in chapter II.

The cultivated shrimps were both males and females as it is usually done in shrimp farming. The animal sex has not significant influence in the results since it is appropriate for what can be found in aquaculture industrial setups.

## CRediT authorship contribution statement

**Fernando J. Ramírez-Coronel:** Methodology, Investigation, Software, Data curation, Writing – original draft, Visualization. **Edgard Esquer-Miranda:** Methodology, Conceptualization, Writing – original draft, Data curation, Formal analysis, Project administration. **Oscar M. Rodríguez-Elias:** Supervision, Conceptualization, Writing – review & editing, Resources, Funding acquisition, Project administration. **Pedro García-Hinostro:** Data curation, Resources. **Guadalupe César Parra-Salazar:** Data curation, Resources.

## Data Availability

A Litopenaeus vannamei shrimp dataset with images and corresponding weight-size measurements for the development of artificial intelligence-based biomass estimation and organism detection algorithms (Original data) (Mendeley Data).
